# Zebra Mussel Holobionts Fix and Recycle Nitrogen in Lagoon Sediments

**DOI:** 10.3389/fmicb.2020.610269

**Published:** 2021-01-19

**Authors:** Ugo Marzocchi, Stefano Bonaglia, Anastasija Zaiko, Grazia M. Quero, Irma Vybernaite-Lubiene, Tobia Politi, Aurelija Samuiloviene, Mindaugas Zilius, Marco Bartoli, Ulisse Cardini

**Affiliations:** ^1^Integrative Marine Ecology Department, Stazione Zoologica Anton Dohrn, National Institute of Marine Biology, Ecology and Biotechnology, Naples, Italy; ^2^Marine Research Institute, Klaipėda University, Klaipėda, Lithuania; ^3^Center for Water Technology (WATEC), Department of Biology, Aarhus University, Aarhus, Denmark; ^4^Department of Ecology, Environment and Plant Sciences, Stockholm University, Stockholm, Sweden; ^5^Nordcee, Department of Biology, University of Southern Denmark, Odense, Denmark; ^6^Department of Marine Sciences, University of Gothenburg, Gothenburg, Sweden; ^7^Coastal and Freshwater Group, Cawthron Institute, Nelson, New Zealand; ^8^Institute of Marine Science, University of Auckland, Auckland, New Zealand; ^9^Institute for Biological Resources and Marine Biotechnologies, National Research Council of Italy, Ancona, Italy; ^10^Department of Life Sciences and Biotechnology, University of Ferrara, Ferrara, Italy; ^11^Department of Chemistry, Life science and Environmental Sustainability, Parma University, Parma, Italy

**Keywords:** *Dreissena polymorpha*, nitrogen, denitrification, DNRA, nitrogen fixation, *nifH*, Curonian Lagoon

## Abstract

Bivalves are ubiquitous filter-feeders able to alter ecosystems functions. Their impact on nitrogen (N) cycling is commonly related to their filter-feeding activity, biodeposition, and excretion. A so far understudied impact is linked to the metabolism of the associated microbiome that together with the host constitute the mussel’s holobiont. Here we investigated how colonies of the invasive zebra mussel (*Dreissena polymorpha*) alter benthic N cycling in the shallow water sediment of the largest European lagoon (the Curonian Lagoon). A set of incubations was conducted to quantify the holobiont’s impact and to quantitatively compare it with the indirect influence of the mussel on sedimentary N transformations. Zebra mussels primarily enhanced the recycling of N to the water column by releasing mineralized algal biomass in the form of ammonium and by stimulating dissimilatory nitrate reduction to ammonium (DNRA). Notably, however, not only denitrification and DNRA, but also dinitrogen (N_2_) fixation was measured in association with the holobiont. The diazotrophic community of the holobiont diverged substantially from that of the water column, suggesting a unique niche for N_2_ fixation associated with the mussels. At the densities reported in the lagoon, mussel-associated N_2_ fixation may account for a substantial (and so far, overlooked) source of bioavailable N. Our findings contribute to improve our understanding on the ecosystem-level impact of zebra mussel, and potentially, of its ability to adapt to and colonize oligotrophic environments.

## Introduction

Microbial symbionts may drive speciation and evolution (Shropshire and Bordenstein, 2016), but their relevance in organismal ecology has only recently gained widespread recognition (Dittami et al., 2020). Huge progress has been made in this research field thanks to rapidly advancing molecular tools (Petersen and Osvatic, 2018). However, molecular methods alone cannot overcome the major challenge of understanding how host-microbe associations, otherwise known as holobionts (Bordenstein and Theis, 2015), contribute to the functioning of the ecosystems they inhabit (see nested ecosystem concept – Pita et al., 2018). Interdisciplinary approaches combining molecular and geochemical investigations are thus urgently needed to investigate the role of complex and diverse host-microbe associations *in natura* (Petersen and Osvatic, 2018; Beinart, 2019). Historically, most ecological research into biological invasions has focused on detrimental species interactions such as predation and competition. However, microbial associates may play an important role by facilitating niche adaptations and allowing their host to occupy otherwise inaccessible habitats (Shapira, 2016). Recent research shows that associations between bivalves and bacteria are paramount in regulating benthic biogeochemical processes (Smyth et al., 2013; Benelli et al., 2017; Bonaglia et al., 2017; Cardini et al., 2019), with microbes contributing to the metabolic potential and impact of the holobiont, in particular concerning carbon (C) and nitrogen (N) cycling (Petersen et al., 2016; Arfken et al., 2017; Konig et al., 2017). Still, little is known on microbiomes of invasive bivalve holobionts and their role in phenotypic plasticity and colonization potential of the invader, and ultimately its ecosystem-level impact (e.g., alteration of biogeochemical processes).

Zebra mussels (*Dreissena polymorpha*, Pallas 1771) are filter-feeding bivalves native to the Ponto-Caspian region, which successfully invaded several regions in Europe and North America, where they significantly altered community structure and ecosystem functioning (Strayer et al., 1999). Their rapid colonization rates together with proficient filter-feeding activity have been linked with the decline in chlorophyll-a, and increase in water transparency and total phosphorous (P) (Caraco et al., 1997), which may result in an overall shift of the trophic state of the colonized freshwater ecosystems (Kumar et al., 2016). The impact of zebra mussel on N cycling is manifold and includes enhanced release of ammonium (NH_4_^+^) from digested algal biomass (Lavrentyev et al., 2000), stimulation of benthic nitrification (Bruesewitz et al., 2008) and denitrification (Bruesewitz et al., 2006), and release of P to the water column (Benelli et al., 2019) potentially stimulating pelagic dinitrogen (N_2_) fixation. The nature and extent of such impacts may however be seasonal (Bruesewitz et al., 2006) and depend upon intrinsic features of the water body such as morphometry (Higgins and Zanden, 2010) and sediment organic matter content. An additional level of complexity in unraveling the overall impact of zebra mussel on N cycling is the distinction between its ability to alter key microbial transformation indirectly (via stimulating the activity of pelagic and benthic communities) and directly, via the hosted microbiome (e.g., Svenningsen et al., 2012). Although the indirect impact has been extensively documented, the role of its microbiome remains largely unexplored both in terms of metabolic repertoire and magnitude of the N transformations. Unraveling the diverse impacts of zebra mussel on nutrient cycling is pivotal to achieve a comprehensive understanding of its invasiveness and role as ecosystem engineer.

In this study, a combination of biogeochemical and molecular approaches was employed to investigate the impact of zebra mussel on N cycling in the sediment of the largest European lagoon (Curonian Lagoon, SE Baltic Sea). Both a “benthic community” (i.e., intact sediment + zebra mussel colony) and a “holobiont” (i.e., zebra mussel alone) incubations were conducted to quantitatively assess the effect of the zebra mussel holobiont on N cycling and to discern it from its effect on sediment processes.

## Materials and Methods

### Site Description and Samples Collection

Sediment and zebra mussel specimens were collected in June 2018 at a fine-sand site (median grain size 0.238 mm) from a shallow area (1.2 m depth) of the oligohaline Curonian Lagoon (55°20′25.9″N, 21°11′24.4″E). The Curonian Lagoon, is a micro-tidal, low-energy system, characterized by a reduced vertical mixing in particularly in the summer months when the discharge from tributaries and wind intensity are at minimum (Ferrarin et al., 2008; Mezine et al., 2019). At the time of sampling, water temperature was 22.5°C, salinity was 0.2, concentration of dissolved organic and inorganic nitrogen (i.e., DON and DIN) was 57.2 ± 0.7 (Mean ± SEM) and 1.8 ± 0.1 μM, respectively. Height intact cores were collected by hand using Plexiglas liners (i.d.: 8.4 cm, length: 30 cm). Four cores included sediments with an overlying colony of zebra mussels and four cores included bare sediments without mussels or other visible macrofauna. Each liner contained approximately 10 cm of sediment overlaid by 16 cm of water. Additional *in situ* water and zebra mussel specimens were collected for single animal incubations and molecular analyses (see details below). Within 1 h, the samples were transported to the laboratory in cool box filled with *in situ* water. At the laboratory, intact cores were submerged overnight in a temperature-controlled tank (23 ± 0.2°C, 200 L) containing unfiltered, aerated *in situ* water. Homogeneous water conditions were kept in each core via magnetic stirring bar (40 rpm). The following day, intact sediment cores with and without mussel colonies were incubated to assess the impact of zebra mussels on (i) sediment nutrients and oxygen fluxes, and subsequently on (ii) nitrate (NO_3_^–^) reduction processes (benthic community). A second set of incubations was conducted to assess the diversity and relevance of N transformations associated with zebra mussel specimens and their microbiome (holobiont incubations).

### Benthic Community Incubations

After a preincubation of 15 h, the water in the tank was partly renewed. Thereafter, the top of each core was sealed with a Plexiglas lid without leaving a head-space and net fluxes of O_2_, DIN (i.e., NH_4_^+^, NO_3_^–^, NO_2_^–^), DON, and phosphate (PO_4_^3–^) between the benthic compartment and the water were measured in dark, while keeping the water stirring on, as described in Samuiloviene et al. (2019). Incubations lasted for less than 4 h to limit the change in water column O_2_ concentration to ≤20% as this is a prerequisite to maintain a linear rate of change in nutrients concentration over time (Dalsgaard et al., 2000). Oxygen concentration was monitored throughout the incubation with an optical O_2_ meter (FireStingO2, PyroScience GmbH). At the start and at the end of each incubation, 30 mL of water were collected from each core, filtered (Frisenette GF/F filters) and stored into 12 mL Polyethylene vials for later determination of DIN. An additional 40 mL aliquot was filtered into a 20 mL glass vials for DON and PO_4_^3–^ determination. Water samples were stored frozen (−20°C) until analyses.

Following the flux measurements, microcosms were left submerged with the top open overnight before starting the NO_3_^–^ reduction [i.e., denitrification and dissimilatory nitrate reduction to ammonium (DNRA)] measurements via ^15^NO_3_^–^ tracer as described by Dalsgaard et al. (2000). Briefly, ^15^NO_3_^–^ was added to the water of each core from a stock solution (20 mM, 99 atom % Na^15^NO_3_; Sigma-Aldrich) to a final ^15^N enrichment of approximately 60% (^14+15^NO_3_^–^ concentration 6.9 μM). The cores were then closed and incubated for 1.5–3 h in the dark. At the end of the incubation, the mussels were removed, and the water and the sediment were gently mixed to a slurry. Thereafter, 20 mL aliquots of the slurry were transferred into 12 mL exetainers (Labco Ltd.) and 200 μL of 7 M ZnCl_2_ were added to stop microbial activity. An additional 40 mL subsample was collected for ^15^NH_4_^+^ determination. Rates of total denitrification (D_tot_) and its components i.e., denitrification of NO_3_^–^ from the water (D_w_) and denitrification coupled to nitrification (D_n_), were calculated from the fluxes of ^29^N_2_ and ^30^N_2_ according to Nielsen (1992). Overestimation of denitrification due to anaerobic ammonium oxidation (anammox) (Risgaard-Petersen et al., 2003) was assumed negligible, since anammox has been previously reported to be marginal in the lagoon sediment (Zilius, 2011). Rates of DNRA were calculated from the ^15^NH_4_^+^ production, D_tot_, and denitrification of ^15^NO_3_^–^ as in Risgaard-Petersen and Rysgaard (1995). At the end of the incubation, sediment from all cores was carefully sieved (0.5 mm mesh-size) to assess the mussel density and to determine their shell-free dry weight (SFDW) after drying the soft tissue at 60°C to a constant weight.

Inorganic nutrient (i.e., NO_x_^–^, NO_2_^–^, NH_4_^+^, PO_4_^3–^) concentrations were measured with a 5-channel continuous flow analyzer (San^++^, Skalar) using standard colorimetric methods (Grasshoff et al., 1983). Nitrate concentration was calculated as the difference between NO_x_^–^ and NO_2_^–^. Total dissolved nitrogen (TDN) was analyzed by the high temperature (680°C) combustion, catalytic oxidation/NDIR method using a Shimadzu TOC 5000 analyzer with a TN module. Dissolved organic nitrogen (DON) was calculated as difference between TDN and DIN. Samples for ^29^N_2_ and ^30^N_2_ were analyzed by gas chromatography-isotopic ratio mass spectrometry (GC-IRMS, Thermo Delta V Plus). Samples for ^15^NH_4_^+^ were analyzed by the same technique (GC-IRMS) after conversion of NH_4_^+^ to N_2_ by the addition of alkaline hypobromite (Warembourg, 1993).

### Zebra Mussel Holobiont Incubations

To determine rates of N transformation (i.e., denitrification, DNRA, anammox, and N_2_-fixation), associated with the zebra mussel holobiont, a series of ^15^N isotope incubations were carried out with individual specimens in the absence of sediment. Prior to the incubation, the biofilm on the mussels’ shell was carefully removed using a toothbrush and mussels were then rinsed in 0.2 μm double-filtered water. Incubations were performed in bottom-capped Plexiglas cylindrical microcosms (total volume 227 ± 3 mL). The microcosms were filled with 0.2 μm double-filtered aerated *in situ* water amended with ^15^N tracers (see the details below). A stirring magnet allowed for continuous water mixing (40 rpm) during the incubation. Microcosms were capped with gas-tight lids provided with two sampling ports for sample collection and water replacement.

#### Nitrate Reduction

Rates of denitrification, DNRA and anammox were estimated following the revised isotope-pairing technique (r-IPT) (Thamdrup and Dalsgaard, 2002; Risgaard-Petersen et al., 2003). Three treatments were prepared: (1) low ^15^NO_3_^–^ addition (final concentration 6.2 μM), (2) high ^15^NO_3_^–^ addition (final concentration 19.1 μM) and (3) ^15^NH_4_^+^ + ^14^NO_3_^–^ (final concentration 6.3 + 5.3 μM, respectively). Treatments 1 and 2 were used to measure rates of denitrification and DNRA. The different tracer concentrations in treatments 1 and 2 allowed to validate the main assumption of IPT, (i.e., tracer concentration-independency of rates). Treatment 3 allowed to measure anammox. Each treatment included five microcosms: four containing one mussel and one control with filtered water only. To calculate the degree of isotopic enrichment, water samples for NH_4_^+^ and NO_3_^–^ analysis were collected prior and after to the isotope addition. Microcosms were incubated in the dark for 8 h at 23 ± 0.3°C. Every 3 h aliquots were subsampled from each replicate/control, transferred into 12 mL exetainers (Labco, United Kingdom) and poisoned with 200 μL of 7 M ZnCl_2_ for later N_2_ and NH_4_^+^ isotopic determination as described above. Significance of the increase in 15N species (i.e., ^29^N_2_, ^30^N_2_, and ^15^NH_4_^+^) over time was tested via regression analysis using the whole datasets (including all data points) for denitrification (*p* < 0.05) and DNRA (*p* < 0.10), respectively. Production rates were calculated from single incubations (time series) and normalized per grams of biomass (SFDW).

#### N_2_ Fixation

To determine rates of N_2_ fixation, a stock solution of ^30^N_2_-enriched filtered water was prepared using a modified version of the protocol described in Klawonn et al. (2015) (see Supplementary Material). Before starting the incubation, the stock solution was gently transferred into four microcosms to minimize gas exchange with the atmosphere. After the mussels were added, the top lids were closed and incubated in the dark for 12 h. Four additional microcosms were prepared and incubated as above but with unlabeled water to serve as a control for isotopic contamination. At the end of the incubation, the mussels were collected and dissected for SFDW determination. Mussel tissues were then stored at −20°C for later ^15^N incorporation analysis. In addition, ten non-incubated specimens were dissected and store as above for later determination of the natural ^15^N/^14^N ratios. Prior to the isotopic analysis, mussels’ tissues were freeze-dried for 48 h, ground to fine powder and weighed into tin capsules. Samples were analyzed for N elemental composition (%) and isotope ratios (δ^15^N) by continuous flow isotope ratio mass spectrometry (IRMS, Isoprime, GV Instruments Ltd.) coupled with elemental analyzer (Costech Instruments). ^15^N_2_ incorporation rates were calculated as in Montoya et al. (1996). ^15^N_2_ incorporation was considered significant for those samples that showed an atom % excess that was higher than two times the standard deviation of the atom % of the unlabeled samples.

### Molecular Analyses of the Prokaryotic Communities

#### Nucleic Acids Extraction and Sequencing

Analysis of 16S rRNA gene and of the *nifH* gene expression were conducted to characterize the N_2_-fixing community in the mussel’s microbiome and its possible relationship with the N_2_-fixing community in the water via filter-feeding activity. Nucleic acids were extracted from the soft tissue of zebra mussels (from in the holobiont incubation) and from the suspended material from *in situ* water sample. Suspended material was size-fractioned in two size groups, i.e., >10 μm, and 0.22–10 μm (from here on referred to as *large* and *small* fraction, respectively) by step-wise filtration of the water as described in Zilius et al. (2020). All samples were collected and analyzed in triplicates. Samples was snap-frozen in liquid nitrogen and stored at −80°C until DNA and RNA extraction. DNA was extracted using the QIAamp Fast DNA Stool Mini Kit (QIAGEN) with increased lysis temperature to 90°C to improve the bacterial cell rupture. RNA was extracted using the RNAeasy Mini Kit (QIAGEN) as in Zilius et al. (2020) and treated with TURBO DNase (Invitrogen). Complementary DNA (cDNA) was synthesized using SuperScriptIII Reverse Transcriptase (Invitrogen), RNaseOUT Ribonuclease Inhibitor (Invitrogen) and random primers. Two negative controls without either reverse transcriptase or RNA were included to assess the potential contamination with residual DNA. Partial 16S rRNA gene sequences were amplified using primer pair Probio_Uni (5′-CCTACGGGRSGCAGCAG-3′) and Probio_Rev (5′-ATTACCGCGGCTGCT-3′), targeting the V3 region of the 16S rRNA gene sequence as described by Milani et al. (2013). High-throughput sequencing was performed at the DNA sequencing facility of GenProbio srl^1^ on an Illumina^TM^ MiSeq with the length of 250 × 2 bp, according to the protocol reported in Milani et al. (2013).

The cDNA-based amplification of *nifH* gene was performed using a nested PCR approach (Zehr and Turner, 2001) with nifH3 and nifH4 primers in the first PCR round followed by second amplification round with nifH1 and nifH2 primers with Illumina indices. Nested PCR conditions were set as in Zilius et al. (2020). Only single bands of appropriate size (359 bp) were detected after the second round of amplification. PCR products were purified from the gel (Thermo Scientific GeneJET Gel Extraction Kit), quantified (Qubit 3.0 Fluorometer) and the sequencing library was constructed following the two-step tailed PCR amplicon procedure, as described in Kozich et al. (2013). Paired-end sequences (2 × 250 bp) were generated on an Illumina MiSeq^®^ instrument using the TruSeq^®^ SBS kit. Sequence data were automatically demultiplexed using MiSeq Reporter (v2), and forward and reverse reads were assigned to samples. Raw sequence data for the 16S rRNA and *nifH* dataset were bioinformatically processed as described in Zilius et al. (2020). Briefly, primers from the raw sequence reads (with Illumina adapters removed by sequencing facility) were trimmed using cutadapt v2.10 (Martin, 2011), with no primer mismatch allowed. The bioinformatics pipeline was run using DADA2 package implemented in R (Callahan et al., 2016). Quality filtering and denoising of the trimmed fastq files were performed using the following parameters: “truncLen = c(150,150), maxEE = c(2,6), truncQ = 2, ndmaxN = 0.” Singletons were discarded, and the remaining paired-end reads were merged with a minimum overlap of 65 bp and 1 mismatch allowed in the overlap region. Chimera removal was performed using the default (consensus) method and the resulting de-noised amplicon sequence variants (ASV) were used for taxonomic classification against the SILVA 132 database for 16S rRNA (Quast et al., 2013) and *nifH* Sequence Database (Gaby and Buckley, 2014). Sequences are available in the NCBI/SRA database under accession number PRJNA658818.

#### Statistical Analyses on the Sequencing Data

The de-noised ASV tables and assigned taxonomy of *nifH* and 16S datasets were imported in RStudio (R Core Team, 2018), combined into two phyloseq objects and processed for data analysis (McMurdie and Holmes, 2013). Rarefaction curves were plotted for both 16S and *nifH* datasets using ggrare function in R (package *ranacapa*; Kandlikar et al., 2018). ASV tables for 16S and *nifH* were rarefied to the lowest number of reads (9,395 and 51,180 for the 16S and *nifH* dataset, respectively) (R package *phyloseq*). For 16S, alpha diversity indices (ASV richness, Shannon index, Simpson index and Pielou’s evenness) were calculated using the R package *vegan* (Oksanen et al., 2019) and number of shared ASVs visualized with Venn diagram (package *venn*; Dusa, 2020). For *nifH*, only ASV richness was calculated. A Kruskal–Wallis test was used to assess differences in 16S and *nifH* ASV richness between water and zebra mussel samples (R package *phyloseq*). Differences in community composition were assessed using the Analysis of Similarity (ANOSIM) based on a Bray–Curtis similarity matrix implemented in *vegan*. PCoA was performed to explore and visualize similarities among the different samples, basing on the same Bray–Curtis similarity matrix, for both the 16S and *nifH* genes datasets. A heatmap with hierarchical clustering was plotted to visualize differences in the abundance of the top 70 16S rRNA gene ASVs (>0.01% across the dataset) using the R packages *Heatplus* (Ploner, 2020), *ggplot2* (Wickham, 2009), and *vegan*. Finally, to gain further information on the identity of unknown Bacteria and unknown Firmicutes identified in the *nifH* dataset for zebra mussel samples, blastn (search in nucleotide databases using a nucleotide query) and blastx (search in protein databases using a translated nucleotide query) (Altschul et al., 1990) analyses were performed against GenBank database (released version 237, May 2020).

## Results

### Respiration and Nutrient Fluxes in Benthic Community Incubations

**FIGURE 1:**
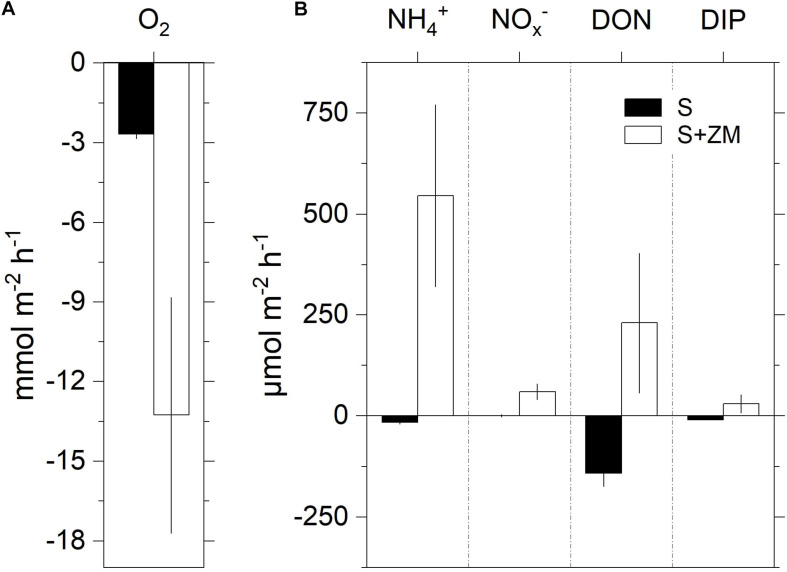
Net fluxes of oxygen **(A)** and nutrients **(B)** at the sediment-water interface during the incubation of intact sediment cores with colonies of zebra mussel (S + ZM) and bare sediments (S). Data are shown as mean values ± standard error (*n* = 3–5).

Mussel total biomass varied between 0.6 and 1.0 g (_*SFDW*_) per core, corresponding to an average areal biomass (±SD) of 134 ± 38 g (_*SFDW*_) m^–2^ and a density of 30–64 mussels per colony. Mean benthic O_2_ consumption was fivefold higher in the presence of the mussels (S + ZM) compared to the bare sediment (S) (Figure 1A). Bare sediment was a net sink for all the measured nutrients (Figure 1B). The presence of mussels reversed the fluxes resulting in the net efflux of all the analyzed species. Net NH_4_^+^ flux accounted for the largest share of the whole DIN efflux. For all measured parameters the difference between net fluxes in S + ZM and S was significant (Mann–Whitney U test, *p* < 0.05).

**FIGURE 2:**
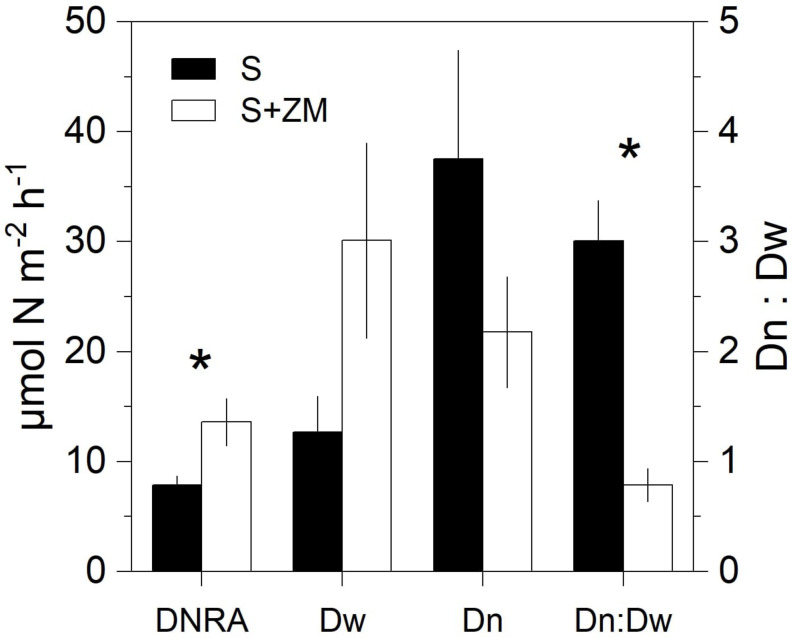
Rates of DNRA and denitrification (showed as partitioned in its two components D_*w*_ and D_*n*_ and their ratio) in incubations of intact sediment cores with colonies of zebra mussel (S + ZM) and bare sediments (S). Data are shown as mean values ± standard error (*n* = 4). Asterisks indicate significant differences (*p* < 0.05, Mann–Whitney U test, *n* = 8).

Rates of DNRA were significantly higher (+72%) in cores with mussels compared to the bare sediment (Mann–Whitney U test, *p* < 0.03) (Figure 2). D_*w*_ tended to be higher in the presence of the mussels compared to the bare sediment (Mann–Whitney U test, *p* = 0.06). D_*n*_ showed an opposite trend with lower rates in the presence of mussels, although the difference was not significant (Mann–Whitney U test, *p* = 0.23). The D_*n*_:D_*w*_ ratio was lower with the mussels compared to the bare sediment (Mann–Whitney U test, *p* < 0.02). Overall denitrification (D_*n*_ + D_*w*_) was unaltered by the presence of the mussel (Mann–Whitney U test, *p* = 0.66).

### N Cycling Associated With the Zebra Mussel Holobiont

**FIGURE 3:**
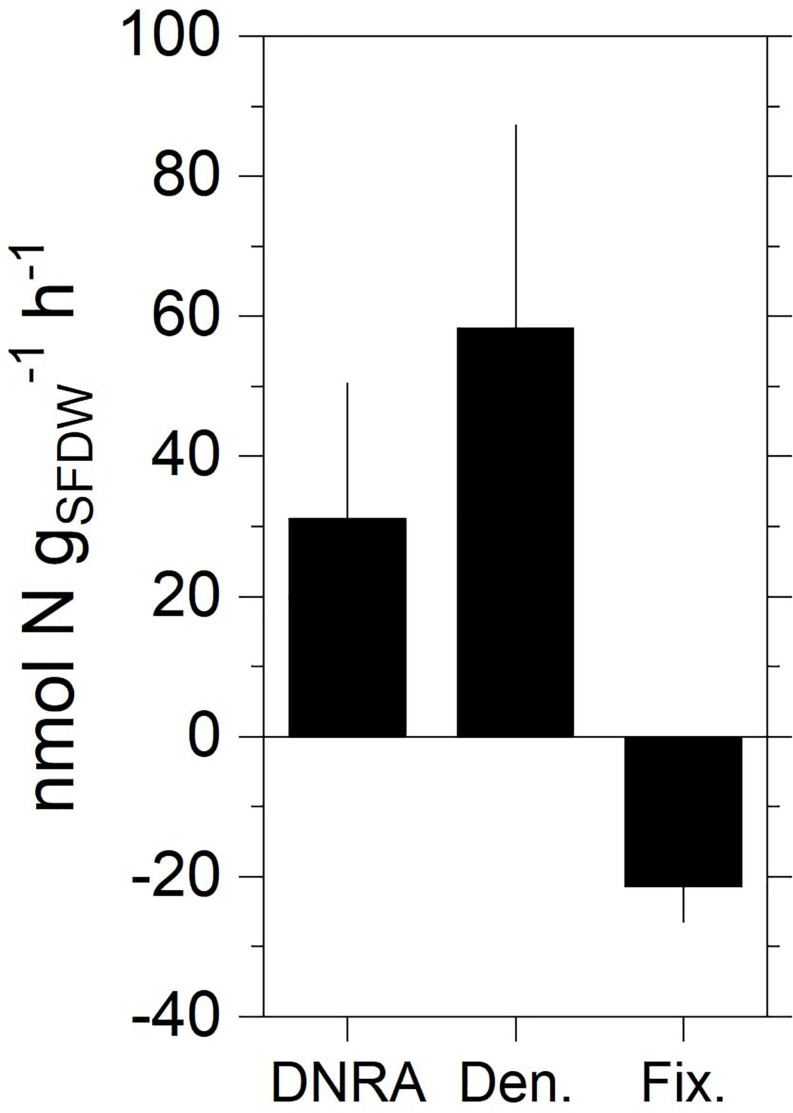
Rates of DNRA, total denitrification, and N_2_ fixation measured in the holobiont incubations with specimens of zebra mussel. Data shown are mean values ± standard error (*n* = 10 for DNRA and denitrification; *n* = 4 for fixation).

The average biomass of the incubated specimens was 37 ± 10 (SD) mg (_*SFDW*_). Regression analysis showed a significant increase in ^15^NH_4_^+^ and ^15^N-N_2_ (i.e., ^29^N_2_, ^30^N_2_) in the DNRA and denitrification incubations, respectively (Supplementary Table 1). Biomass-normalized rates of DNRA spanned between zero and 192 nmol N g _(SFDW)_^–1^ h^–1^ (average ± SEM, 31.2 ± 19.3 nmol N g _(SFDW)_^–1^ h^–1^) (Figure 3). Rates of denitrification ranged between zero and 260 nmol N g (_*SF*__*DW*_)^–1^ h^–1^ (average ± SEM, 58.4 ± 28.9 nmol N g _(SFDW)_^–1^ h^–1^). No anammox activity was detected within the timespan of the incubation (results not shown). N_2_ fixation was detected in all tested animals (Supplementary Table 2) at rates ranging between 7.8 and 30 nmol N-N_2_ g (_*SF*__*DW*_)^–1^ h^–1^ (average ± SEM, 21.9 ± 4.5 nmol N g _(SFDW)_^–1^ h^–1^). On average, under our experimental conditions, N_2_ fixation was equal to 37% of the denitrification rate.

### Water Column and Mussel-Associated Microbial Communities

After denoising and eukaryote sequence removal, the complete 16S rDNA dataset comprised 447,071 good quality sequence reads from the nine analyzed samples representing 2,705 bacterial ASVs. Rarefaction curves (Supplementary Figure 1) evidenced that the sequencing effort was sufficient to describe bacterial diversity. The normalized ASV richness (after rarefying the sequences at an even depth of 9,395) was significantly higher in zebra mussel compared to both size fractions of the water samples (Kruskal–Wallis, *p* < 0.01) (Supplementary Figure 2). Shannon and Simpson diversity and Pielou’s evenness indices showed significantly higher values in the large compared to the small fraction of water samples. Shannon diversity tended to be higher in zebra mussel samples, although no significant difference with water samples was observed (*p* < 0.05). Zebra mussel and water samples (small and large fractions) shared 13.3% of the detected ASVs (*n* = 359), while 1,074 ASVs (39.7%) were exclusively associated with zebra mussels (Supplementary Figure 3).

**FIGURE 4:**
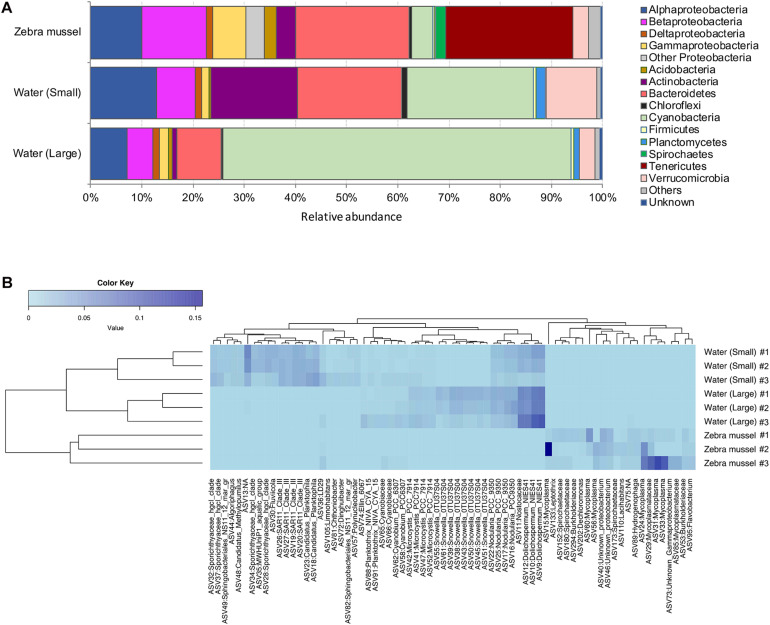
**(A)** Bacterial community composition (based on the 16S rRNA gene) at the phylum and class (only for Proteobacteria) levels in zebra mussels and water column particulate samples of large (>10 μm) and small (0.22–10 μm) size fractions. Phyla and classes with an average relative abundance <0.5% across all samples were aggregated and reported as “Others.” “Unknown” includes all those reads not matching any known bacterial taxonomy. **(B)** Heatmap summarizing the relative abundances of the top 71 ASVs in the analyzed samples.

The three types of samples showed distinct relative abundances of major prokaryotic taxa (Figure 4A) (ANOSIM, global *R* = 0.88; *p* < 0.01) and grouped separately when analyzed by PCoA (Supplementary Figure 4A). Zebra mussels were characterized by high abundances of Tenericutes (average, 25%), that were basically undetectable in water samples. Beta- (average, 12.8%) and Gammaproteobacteria (average, 6.5%), and Bacteroidetes (average, 22.5%) accounted for a considerable fraction in zebra mussel samples, while a general lower presence of Cyanobacteria (average, 4.3%) and Actinobacteria (average, 3.8%) was observed in comparison to both types of water samples. The Tenericutes phylum was almost entirely represented by members of the genus *Mycoplasma*, with relative abundances up to 40% of the overall community. In the large fraction of the water samples, Cyanobacteria clearly dominated the community (average, 67.8%), while in the small fraction, a more even community structure was observed, represented by Cyanobacteria (average, 24.7%), Bacteroidetes (average, 20.6%), and Actinobacteria (average, 16.9%), Alpha- (12.6%) and Betaproteobacteria (7.8%).

The heatmap visualization of the most abundant (>0.1%) ASVs, supported the taxonomic differentiation of the microbial communities between two water fractions and zebra mussel samples (Figure 4B). In particular, the zebra mussel microbial community was characterized by a pool of taxa mainly including *Mycoplasma* and Mycoplasmataceae (eight ASVs, average, 3% of zebra mussel reads), Spirochaetaceae (three ASVs, average 0.5%), Burkholderiaceae (two ASVs, average 0.6%), *Lacihabitans* (1), Sphingomonadaceae (1), *Dechloromonas* (1), *Hydrogenophaga* (1), *Flavobacterium* (1), and three unidentified Proteobacteria. Water samples were characterized by the presence of numerous Cyanobacteria and freshwater taxa (e.g., *Limnohabitans*, *Polynucleobacter*).

### Water Column and Mussel-Associated Active Diazotrophic Communities

**FIGURE 5:**
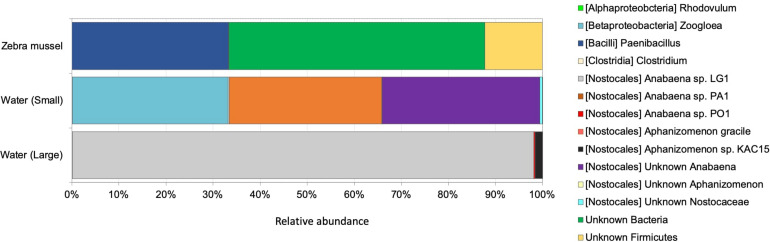
Diazotrophic community composition based on *nifH* transcripts diversity in zebra mussel and water column particulate samples of large (>10 μm) and small (0.22–10 μm) size fractions. Data are shown as average of three replicates.

The *nifH* dataset comprised a total of 2,045,435 good quality reads (on average, 227,270 reads per sample, ranging from 51,180 in a zebra mussel sample to 389,159 in a large fraction water sample), representing 360 *nifH* ASVs. Rarefaction curves for *nifH* (Supplementary Figure 5) confirmed the adequate diversity coverage at the attained sequencing depth. After rarefying at 51,180 sequence depth, a total of 344 ASVs were retained for the downstream analyses. No statistically significant difference in ASV richness was observed between the two water fractions and zebra mussel samples (Kruskal–Wallis rank sum test, *p* = 0.707) (Supplementary Figure 6). Diazotrophic communities significantly differed among the three type of samples (Figure 5; ANOSIM, global *R* = 0.543; *p* = 0.006) and grouped separately when analyzed by PCoA (Supplementary Figure 4B). In zebra mussel samples, the diazotrophic community was dominated by a large fraction of unknown Bacteria (54.4%), followed by *Paenibacillus* (33.2%) and a smaller fraction of unknown Firmicutes (12.2%). Interestingly, none of these groups were detected in water samples, which were almost completely dominated by Nostocales (83.4%) and *Zoogloea* (16.6%). More specifically, the small fraction of the water samples was dominated by *Anabaena* (66.2%) and *Zoogloea* (33.1%), while the large fraction of water samples by *Anabaena* (98.4%).

After blastn analyses, all ASV sequences identified as unknown Firmicutes matched with *nifH* sequences belonging to Clostridia, although weakly (<79% of similarity, 99% query coverage). Protein sequence similarity search analyses based on translated proteins (i.e., blastx) also indicated, that such ASVs were loosely related to Clostridia (90–92% of similarity, 99% query coverage). Blastn analyses performed on ASV identified as unknown Bacteria matched, although at low similarities (75–82%), mostly with *Azotobacter*, *Paenibacillus*, and Clostridia. Results from blastx indicated that most of the ASVs identified as unknown Bacteria were highly related to Clostridia and *Paenibacillus* (80.5–93.5%). Some of the ASVs, showed similarities up to 91.5% with queries belonging to the phylum Bacteroidetes.

## Discussion

### Impact of Zebra Mussel on Benthic N Cycling

**FIGURE 6:**
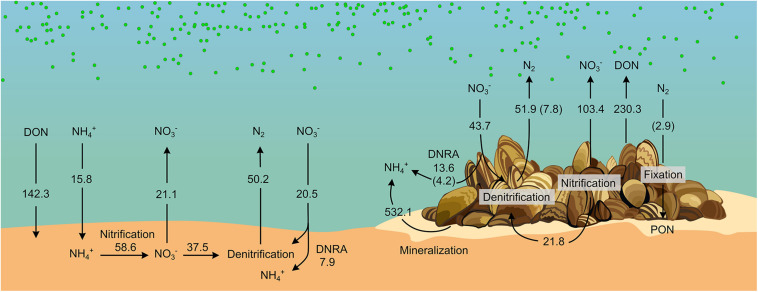
Diagram of N cycling in bare sediments and sediment with zebra mussel colonies. Fluxes (μmol m^− 2^ h^− 1^) were obtained from benthic-community incubations. Contributions from Zebra mussels’ microbiome (displayed within brackets) were obtained from biomass-specific fluxes measured in the holobionts incubation and scaled to the biomass present in the benthic-community incubations. See details on the calculation in Supplementary Material section “Calculations for the Build-Up of the N Diagram Showed in Figure 6.” Drawing by V. Gasiūnaitė.

Although it is well documented that benthic macroinvertebrates alter vital sediment processes such as N turnover through irrigation and bioturbation (e.g., Stief, 2013), animal-bacterial associations and their role on biogeochemical cycling remain largely unresolved. Here, we quantitatively assessed how a model invasive bivalve (the zebra mussel) alters benthic N cycling both directly (e.g., via excretion of DIN and DON) and indirectly via stimulating microbial activity both at the sediment level and through its microbiome. Figure 6 summarizes rates of N fluxes measured in the benthic community incubations, and the biomass-specific rates measured in the holobiont incubations after extrapolation using the densities from the benthic community experiment. As evidenced, the presence of zebra mussels turned the benthic compartment from a sink to a source of all measured nutrients. In particular, NH_4_^+^ was the most prominent dissolved N species released into the water. Enhanced benthic release of NH_4_^+^ has been consistently reported in the presence of zebra mussels (James et al., 1997; Lavrentyev et al., 2000; Conroy et al., 2005; Ruginis et al., 2014) and other filter feeders (Mazouni et al., 1996; Bartoli et al., 2003; Nizzoli et al., 2006). The increase in NH_4_^+^ efflux can be sustained by three mechanisms: (i) mineralized algal biomass from filter-feeding activity of the mussel, (ii) stimulation of DNRA activity, and (iii) inhibition of nitrification due to the colony physical presence and consequent limitation of the O_2_ transport into the sediment (Zaiko et al., 2010). The proximity of the NH_4_^+^: DIP ratio (i.e., 18) to the Redfield ratio calculated from the fluxes in the benthic-community incubations suggests that NH_4_^+^ most likely originates from mineralization of algae biomass by zebra mussel. Accordingly, enhanced rates of DNRA measured in the benthic-community incubations with the mussels could only contribute 1.0% of the enhanced NH_4_^+^ efflux. Net fluxes of NO_3_^–^ in the whole-community incubations suggest a stimulation of nitrification by zebra mussel rather than its inhibition. Assuming that the drop in D_*n*_ measured in the presence of zebra mussel is caused by the suppression of nitrification, the resulting release of NH_4_^+^ would, however, only contribute to 4% of the overall sediment efflux of NH_4_^+^. Our data therefore indicate that the most prominent impact of zebra mussel on DIN dynamics is via the recycling of fixed N through mineralization of pelagic algae and other particulate organic matter either being egested as biodeposits or retained within the mussels’ colony.

The net release of DON in the presence of the mussel might be sustained by the mussels’ egestion/excretion or derive from exudates by settled phytoplankton aggregates within the colony. Recently, it has been shown that other dreissenids excrete dissolved organic matter with relatively low C to N ratio indicating high proportion of organic N compounds (DeVilbiss and Guo, 2017). The biochemical mechanisms at the basis of such DON release, the conditions that promote it, and its environmental relevance remain, however, poorly understood in filter-feeders.

Contrary to what previously reported from the upper Mississippi River (Bruesewitz et al., 2006, 2008) and from a freshwater Lithuanian lake (Ruginis et al., 2014), zebra mussels did not increase overall benthic denitrification in our incubations. Rather, the presence of the mussels altered the balance between D_*w*_ and D_*n*_ favoring the former over the latter. Denitrification was previously reported from zebra mussels holobiont incubations, possibly occurring in the gut (Svenningsen et al., 2012). However, our holobiont incubations showed that such contribution accounted only for 15% of the denitrification rates measured in the benthic-community incubations (Figure 6), suggesting that the impact of zebra mussel on denitrification was mainly indirect (related to the altered sediment microbial activity), rather than via stimulation of NO_3_^–^ reduction in anoxic sections of the animal body.

On the contrary, DNRA activity in the holobiont incubation accounted for a major fraction (i.e., 74% = 4.2 μmol m^–2^ h^–1^, Figure 6) of the increment in DNRA measured in the benthic-community incubation in the presence of the mussels (i.e., +5.7 μmol m^–2^ h^–1^, Figures 2, 6), indicating a dominant effect of the mussels’ microbiome in stimulating DNRA. DNRA bacteria have a competitive advantage over denitrifiers when the organic carbon to NO_3_^–^ ratio is high (Tiedje, 1988). Such conditions are plausibly met in the anoxic section of the mussels’ gut. Accordingly, DNRA to denitrification ratio was higher in holobiont incubations (i.e., 0.58) compared to the benthic-community incubation (i.e., 0.16–0.26), suggesting a relatively more favorable niche for DNRA activity in the animal’s gut compared to the surrounding sedimentary environment.

Increase in NO_3_^–^ efflux and D_*w*_ and simultaneous decrease in D_*n*_ are compatible with the thinning of the sediment oxic layer as due to the accumulation of labile phytodetritus (Marzocchi et al., 2018). Similarly, the biodeposition of labile organic carbon by zebra mussel (in the form of feces and pseudo-feces) has been shown to enhance benthic respiration causing the thinning of the sediment oxic layer (Bruesewitz et al., 2008). Such a decrease of the O_2_ penetration depth shortens the diffusional path for water-NO_3_^–^ to reach the denitrification zone, hence, enhancing D_*w*_. At the same time, the contraction of the oxic portion of the sediment diminishes the sediment volume suitable for nitrification, favoring the diffusion of sediment NH_4_^+^ to the water and thus partially decoupling nitrification and denitrification. Moreover, nitrification activity occurring at shallower depths is expected to favor the diffusion of NO_3_^–^ to the water, further contributing to the decrease in D_*n*_. Assuming that the drop in D_*n*_ is caused by a preferential release of NO_3_^–^ to the water, the so generated NO_3_^–^ would account for 33% of the measured net NO_*x*_^–^ effluxes in the benthic-community incubation. The accumulation of biodeposits by zebra mussel was visually observed during our incubations, providing a plausible, additional, mechanism by which zebra mussel impacts benthic N dynamics via altering the architecture of the habitat.

### N_2_ Fixation by Zebra Mussel Holobionts

This study is the first, to our knowledge, to report N_2_ fixation associated with zebra mussel holobionts. Our incubations show that if unaccounted, this process can lead to 6 and ∼60% overestimation of the benthic community and holobiont-associated net N_2_ fluxes, respectively. At the lagoon level, N_2_ fixation has been traditionally attributed to pelagic cyanobacterial activity (Lesutienė et al., 2014; Bartoli et al., 2018; Zilius et al., 2020) and reported to occur seasonally (spring and winter) in undisturbed sediments (Zilius et al., 2018). Dinitrogen fixation associated with the zebra mussel (and more in general in mussel-colonized sediment) has not been accounted so far in estimations of the lagoon’s N mass balance (Zilius et al., 2018). The zebra mussel is a dominant benthic organism in the Curonian Lagoon sediment where it has been reported at densities ranging between 40 and 57,000 individuals per square meter (median 12,600) (Daunys et al., 2006). Scaled-up to these abundances, N_2_ fixation rates derived from our incubations can account for 0.01 to 19.9 μmol of fixed N m^–2^ h^–1^ (median 4.4 μmol N m^–2^ h^–1^), respectively. In summer, cyanobacterial-driven N_2_ fixation has been reported at rates between 0.9 and 209.4 μmol m^–2^ h^–1^ (median 33.7 μmol^–2^ h^–1^; Zilius et al., 2018). Thus, zebra mussel holobionts could possibly contribute a substantial (and so far disregarded) input of N to the lagoon, offsetting the attenuation of the N load via denitrification, and therefore mitigating summer N limitation of the lagoonal system (Vybernaite-Lubiene et al., 2018). Considering maximum densities of 100,000 individuals per square meters reported from zebra mussel-colonized riverine sediments (Svenningsen et al., 2012), its impact could potentially alter N pathways at a scale significantly exceeding that assumed from our experiments and calculations. Further studies are needed to assess the overall relevance of N_2_ fixation driven by zebra mussels holobionts, its variation under diverse environmental conditions and its seasonal patterns.

The analysis of the microbiome associated with zebra mussels showed that comparatively few ASVs were shared with the waterborne microbial community. The detected high diversity of mussel-associated assemblages (with many taxa not observed in water samples) is consistent with previous findings of specific and diverse bacterial communities associated with bivalves (Lokmer et al., 2016; Cleary and Polonia, 2018; Vezzulli et al., 2018; Mathai et al., 2020). Tenericutes, and more specifically *Mycoplasma*, abundant in the zebra mussel samples, are typical constituents of the core bivalve gut microbiome (Pierce, 2016; Aceves et al., 2018; Pierce and Ward, 2018, 2019), including zebra mussels (Mathai et al., 2020). These obligate cell-associated bacteria are commonly found within a number of eukaryotic hosts and, although previously considered as parasites or even a sign of infection, are now assumed to be involved in mutually beneficial interactions with the host (Fraune and Zimmer, 2008; Holm and Heidelberg, 2016; van de Water et al., 2018). However, no diazotrophic activity has been attributed to any taxa of the Tenericutes phylum (Dos Santos et al., 2012; Albright et al., 2019). On the other hand, the *Flavobacterium* genus, commonly identified in or isolated from bivalves (Pujalte et al., 1999; Aceves et al., 2018; Pierce and Ward, 2019), and fairly abundant in zebra mussel samples from our incubations, includes some species carrying nitrogenase genes. Several studies confirmed the ability of *Flavobacterium* isolates to perform N_2_ fixation, although this has been demonstrated mainly in plants (Giri and Pati, 2004; Kampfer et al., 2015). A number of other potential diazotrophs were detected in zebra mussel samples in our study. Along with Tenericutes, Spirochetes are well-documented common members of bivalve gut microbiome (Margulis and Hinkle, 1992). This taxon dominates the microbiome of other eukaryotic organisms in different environments (Lilburn et al., 2001; van de Water et al., 2016) and has been shown to exhibit diazotrophic activity. Burkholderiaceae are among the most well-known N_2_-fixing bacterial groups in plants (Sprent et al., 2017). Species of this family occupy diverse ecological niches and can be found in soil and water, and in association – even symbiosis – with plants, animals, and fungi (Coenye, 2014). Species of the genus *Leptothrix*, belonging to the Burkholderiaceae family, are commonly found in lakes, lagoons, and swamps, and species of this genus have been studied and isolated as root endophytes in plants (López-López et al., 2010; Li et al., 2011). Finally, members of both *Hydrogenophaga* (i.e., *Hydrogenophaga pseudoflava*) and *Dechloromonas* have shown the ability to fix N_2_ (Willems et al., 1989; Salinero et al., 2009), although this has been observed so far only in plants, and to our knowledge no indications of diazotrophic activity carried out by these taxa have been reported in bivalves.

The diversity of active diazotrophs in zebra mussels, as characterized by *nifH* gene transcription analysis, also differed substantially from that of water samples. Unlike pelagic diazotrophs, which were mainly represented by Cyanobacteria, *nifH* transcript diversity of the mussels was dominated by *Paenibacillus* and other taxa closely related to Clostridia and Bacteroidetes. Such taxa have been previously described as diazotrophs, although, a few evidences have suggested – so far – their association with bivalves. *Paenibacillus* (phylum Firmicutes) is a genus widely known to include N_2_-fixing species in soil, and recent studies highlighted its frequent detection and potential role in N_2_ fixation in aquatic environments (Yu et al., 2018; Pang et al., 2019; Tang et al., 2019). However, to our knowledge, this is the first study reporting its association with benthic invertebrates. On the contrary, Clostridiales have been described as the most frequently detected sequences in the microbiome of Unionidae mussels (Weingarten et al., 2019), which co-exist with zebra mussels in the Curonian Lagoon (Benelli et al., 2019). Besides, Clostridiales are common in the gut microbiome of vertebrates (Colston and Jackson, 2016). In addition, several members of the Clostridiales are euryhaline, may thus perform N_2_ fixation in the wide range of conditions as those found in estuarine environments (Herbert, 1975). Finally, many Bacteroidetes bacteria possess nitrogenase genes, and are thus capable of N_2_ fixation (Inoue et al., 2015); however, to the best of our knowledge, studies reporting associations between bivalves and members of Bacteroidetes and/or describing the role of this taxon in N_2_ fixation in aquatic invertebrates are missing.

## Conclusion

Our results show that zebra mussels favor the recycling of N via algal mineralization and by stimulating DNRA activity both in the sediment and via its microbiome. In addition, the mussels mediate a so far overlooked input of nitrogen via N_2_ fixation. Diazotrophic activity is likely sustained by a unique mussel-associated microbial community, which differs substantially from the N_2_-fixing community in the water column. Further investigations are needed to assess whether the association of zebra mussels with diazotrophs is a transient interaction or a stable symbiosis, as well as potential fluxes of energy and matter between the microbiome and the host. The capability to host diazotrophic bacteria might be particularly advantageous for zebra mussels to facilitate their establishment and spread in nutrient-poor environments and might therefore represent an important factor in determining their high invasiveness and adaptive capacity. It may also provide an advantage in eutrophic estuaries such as the Curonian Lagoon, which typically display pronounced seasonal variations in inorganic N availability.

## Data Availability Statement

The datasets presented in this study can be found in online repositories. The names of the repository/repositories and accession number(s) can be found below: https://www.ncbi.nlm.nih.gov/, PRJNA658818.

## Author Contributions

SB, MB, MZ, and UC conceived and designed the study. UM contributed to the concept. SB, IV-L, TP, AS, MZ, MB, and UC performed the experiments. UM, SB, IV-L, TP, and AS conducted the chemical analyses. UM, SB, and MZ analyzed the geochemical data. AS carried out the nucleic acid extraction and amplicon sequencing. AZ and GQ performed the bioinformatic and statistical analysis on the sequencing data. UM, GQ, and UC wrote the first draft of the manuscript. All authors contributed to draft revisions, read, and approved the final version.

## Conflict of Interest

The authors declare that the research was conducted in the absence of any commercial or financial relationships that could be construed as a potential conflict of interest. The handling editor declared a shared affiliation with one of the authors, SB.

## Footnotes

1 www.genprobio.com
