# Supplementary material for: Zebra Mussel Holobionts Fix and Recycle Nitrogen in Lagoon Sediments
Source: Front Microbiol. 2021 Jan 19;11:610269. doi: 10.3389/fmicb.2020.610269 (PMC7851879; doi:10.3389/fmicb.2020.610269)
Supplement: Supplementary file 1 [file Data_Sheet_1.docx]

**Zebra mussel holobionts fix and recycle nitrogen in lagoon sediments**

**SUPPLEMENTARY INFORMATION**

Ugo Marzocchi^1,2,3*^, Stefano Bonaglia^2,4,5,6^, Anastasija Zaiko^2,7,8^, Grazia Marina Quero^1,9^, Irma Vybernaite-Lubiene^2^, Tobia Politi^2^, Aurelija Samuiloviene^2^, Mindaugas Zilius^2,10^, Marco Bartoli^2,11^, and Ulisse Cardini^1,2*^

^1^ Integrative Marine Ecology Department, Stazione Zoologica Anton Dohrn, National Institute of Marine Biology, Ecology and Biotechnology, Napoli, Italy

^2^ Marine Research Institute, Klaipėda University, Klaipėda, Lithuania

^3^Center for Water Technology (WATEC),Department of Biology, Aarhus University, Aarhus, Denmark

^4^ Department of Ecology, Environment and Plant Sciences, Stockholm University, Stockholm, Sweden

^5^Nordcee, Department of Biology, University of Southern Denmark, Odense, Denmark

^6^Department of Marine Sciences, University of Gothenburg, Box 461, Gothenburg, 40530, Sweden

^7^ Coastal and Freshwater Group, Cawthron Institute, Nelson, New Zealand

^8^Institute of Marine Science, University of Auckland, Auckland, New Zealand

^9^ Institute for Biological Resources and Marine Biotechnologies, National Research Council of Italy (IRBIM-CNR), Ancona, Italy

^10^Department of Life Sciences and Biotechnology, University of Ferrara, Ferrara, Italy

^11^Department of Chemistry, Life science and Environmental Sustainability, Parma University, Parma, Italy

^*^ Correspondence to: [ugomar@au.dk](mailto:ugomar@au.dk) and [ulisse.cardini@szn.it](mailto:ulisse.cardini@szn.it)

1. **MATERIAL AND METHODS**

**1.1 Preparation of the ^30^N_2_ stock solution**

One 2L glass bottle (Shott, Germany) was filled with filtered *in situ* water and sealed with black butyl septa and screw caps, with a hollow needle in the septum to prevent the formation of air bubbles. Thereafter, 10 mL of^30^N_2_ gas (99 atom% ^15^N, Sigma-Aldrich) were injected in the bottle with a second hollow needle in the septum to allow replacement of the liquid and the formation of a ^30^N_2_ head-space. The procedure was repeated with additional 20 mL of gas without the second needle in the septum to create over-pressure within the bottles and aid dissolution of the gas. The so prepared ^30^N_2_ stock solution was shaken for two hours on a table shaker and stored in the dark, upside down, until use.This procedure resulted in a final ^15^N-N-atom% of 48.5%.

**1.2 Calculations for the build-up of the N diagram showed in figure 6**

Fluxes ($J$) showed in figure 6 were calculated fromaverage fluxes of (NO_3_^-^, NH_4_^+^, DON, DNRA, D_w_, D_n_, and D_tot_) measured in the benthic-community incubations (and showed in Fig. 1 and 2), as follows:

| Fluxes in Figure 6 | Fluxes from incubations |  |
| --- | --- | --- |
| Gross$J{NO}_{3}^{-}$ to the water colum | = Net $J{NO}_{3}^{-}$ + D_w_ + DNRA | (Eq. 1) |
| Gross $J{NO}_{3}^{-}$ to the sediment | = D_w_ + DNRA | (Eq. 2) |
| $J{NH}_{4}^{+}$(right panel, bottom arrow) | = Net $J{NH}_{4}^{+}$- DNRA | (Eq. 3) |
| $J{NH}_{4}^{+}$ (right panel, top arrow) | = DNRA | (Eq. 4) |
| $J{NH}_{4}^{+}$ (left panel, arrow to the left) | = Net $J{NH}_{4}^{+}$ | (Eq. 5) |
| $J{NH}_{4}^{+}$ (left panel, arrow to the right) | = DNRA | (Eq. 6) |
| $JDON$ | = Net $JDON$ | (Eq. 7) |
| $JN_{2}$ to the water colum | = D_tot_ | (Eq. 8) |
| Nitrification | = Gross $J{NO}_{3}^{-}$(from eq 1) + D_n_ | (Eq. 9) |

Fluxes within brackets on the right panel are calculated from the biomass-specific fluxes measured in the holobionts incubation (showed in Fig. 3) and scaled to the biomass present in the benthic-community incubations, as follows:

$Jx$(figure 6) = $Jx$ (holobiont incubation) * average biomass in benthic community incubation/ sedimet core surface area.

Where x is either DNRA, Denitrification or N_2_-fixation. The biomass in the benthic community incubation is 0.75 ± 0.29 g dry weight (mean ± St. dev.), and the sediment core surface area is 5.5 x 10^-3^ m^2^.

1. **TABLE AND FIGURES**

**a**

|  |  | *Denitrification* | | | | *DNRA* | |
| --- | --- | --- | --- | --- | --- | --- | --- |
| *Overall* | | *p*^29^N_2_ | *p* | *p*^30^N_2_ | *p* | *p*^15^NH_4_^+^ | *p* |
|  |  | (µmol d^-1^) |  |  |  |  |  |
|  | Controls | -0.004 | 0.75 | 0.014 | 0.277 | 0.004 | 0.73 |
|  | Treatments | **0.014** | **0.04** | **0.216** | **>0.01** | **0.008** | **0.07** |
|  |  |  |  |  |  |  |  |
| *Replicates* | |  |  |  |  |  |  |
| Treatment  **b** | ID | *p*^29^N_2_ | r^2^ | *p*^30^N_2_ | r^2^ | *p*^15^NH_4_^+^ | r^2^ |
|  |  | (µmol d^-1^) |  | (µmol d^-1^) |  | (µmol d^-1^) |  |
| ^15^NO_3_^-^ low | Control | -0.007 | 0.11 | -0.004 | 0.08 | 0.002 | 0.01 |
|  | 1 | 0.000 | 0.00 | 0.000 | 0.03 | 0.005 | 0.04 |
|  | 2 | -0.006 | 0.13 | -0.001 | 0.05 | **0.016** | **0.47** |
|  | 3 | **0.036** | **0.81** | **0.015** | **0.82** | -0.004 | 0.03 |
|  | 4 | **0.058** | **0.93** | **0.012** | **0.73** | **0.014** | **0.34** |
|  | 5 | -0.006 | 0.24 | 0.002 | 0.06 | 0.011 | 0.15 |
| ^15^NO_3_^-^ high | Control | 0.001 | 0.00 | 0.001 | 0.05 | -0.005 | 0.00 |
|  | 1 | **0.021** | **0.98** | **0.010** | **0.76** | 0.000 | 0.00 |
|  | 2 | **0.008** | **0.80** | **0.013** | **0.89** | 0.013 | 0.16 |
|  | 3 | **0.005** | **0.70** | **0.013** | **0.88** | **0.011** | **0.34** |
|  | 4 | -0.001 | 0.02 | 0.001 | 0.02 | -0.002 | 0.05 |
|  | 5 | **0.015** | **0.93** | **0.011** | **0.44** | **0.017** | **0.39** |

**Table S1**.Results of the regression analyses (concentration of ^15^N species vs time) in the holobiontincubation. *Overall* values show the results for Controls and Treatments with combined replicates.In bold are represented values with p< 0.05 for denitrification and p <0.1 for DNRA which were used to indicate significant production of 15N-N2 and 15NH4+, respectively (**a**).*Replicates* values show the results for single incubationsat two ^15^NO_3_^-^ concentrations (low and high).In bold are represented values with r^2^> 0.3, which were used for calculating rates of denitrification (*p*^29^N_2_ and *p*^30^N_2_) and DNRA (*p*^15^NH_4_^+^) (**b**).

|  |  | *Fixation* | |
| --- | --- | --- | --- |
| ID | Replicate | Rate | Excess (atom % ^15^N) :St.dev atom % ^15^N Nat. abundance |
|  |  | (nmol N g_SFDW_^-1^ h^-1^) |  |
| Treatment | 1 | **21.4** | **8.0** |
|  | 2 | **7.8** | **3.4** |
|  | 3 | **27.0** | **12.4** |
|  | 4 | **30.0** | **14.0** |
| Control | 1 | -0.3 | -0.3 |
|  | 2 | -0.4 | -0.6 |
|  | 3 | -0.5 | -0.6 |
| Natural abundance (Av. ± St.dev.) | | 0.37037 ± 0.0002 (n = 10) | |

**Table S2**.N_2_-fixation rates (^15^N net accumulation in biomass) measured in experiments with individual zebra mussels incubated in ^15^N enriched water and controls with unlabeled water. ^15^N_2_ incorporation was considered significant for those samples that showed an atom% excess that was higher than 2 times the standard deviation of the atom% of the untreated samples (Natural abundance).


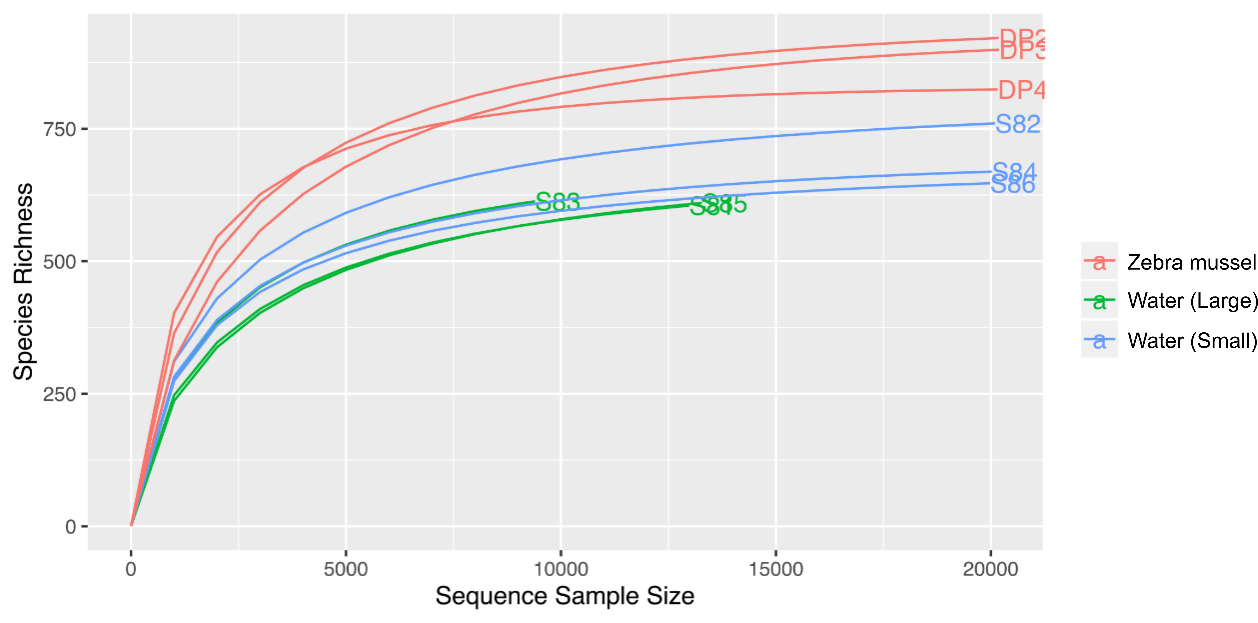


**Figure S1.** Rarefaction curves of the 16S rRNA samples analysed in this study. DP2, DP3 and DP4 refer to zebra mussel samples. Samples S82, S84 and S86 refer to “Large” size fraction of water column particulate matter (> 10 µm); samples S81, S83 and S85 refer to “Small” size fraction of water column particulate matter (0.22- 10 µm).


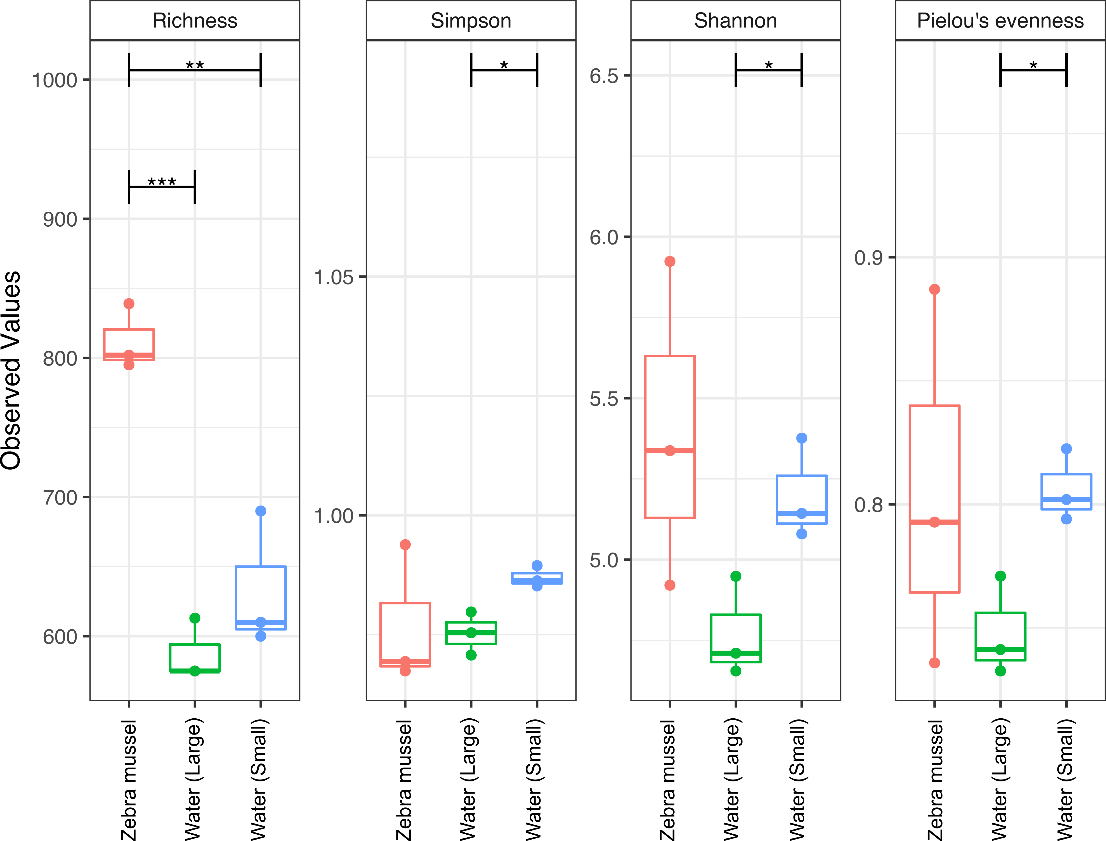


**Figure S2**. Boxplots showing alpha-diversity indices of samples of zebra mussels and water column particulatematter of “Large”(> 10 µm) and “Small” (0.22-10 µm) size. The presence of significant differences between pairs of samples, tested by Kruskal-Wallis test, is also indicated (* = significant for p<0.05; ** = significant for p<0.01; *** = significant for p<0.001).


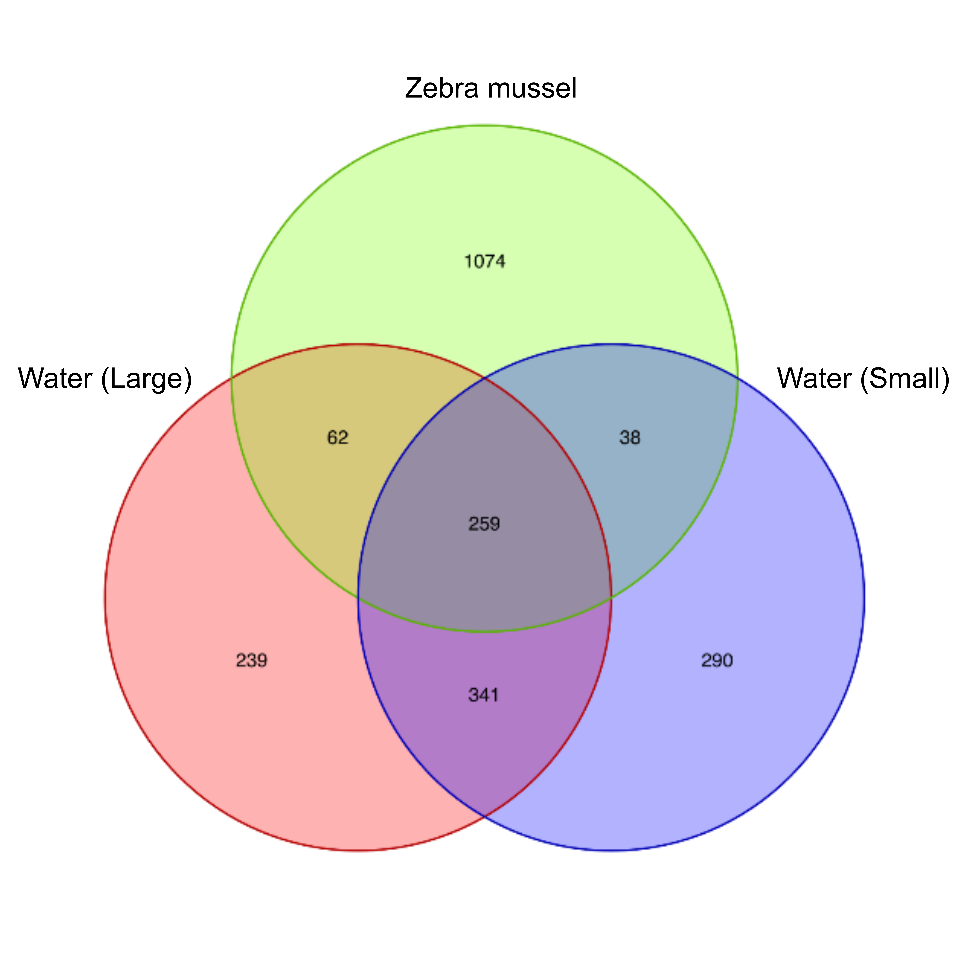


**Figure S3.** Venn diagram showingshared and exclusive ASVs between and among samples of zebra mussel (soft tissues) and water column particulate matter of two size fractions, *i.e.*, > 10 µm (Large) and 0.22-10 µm (Small).


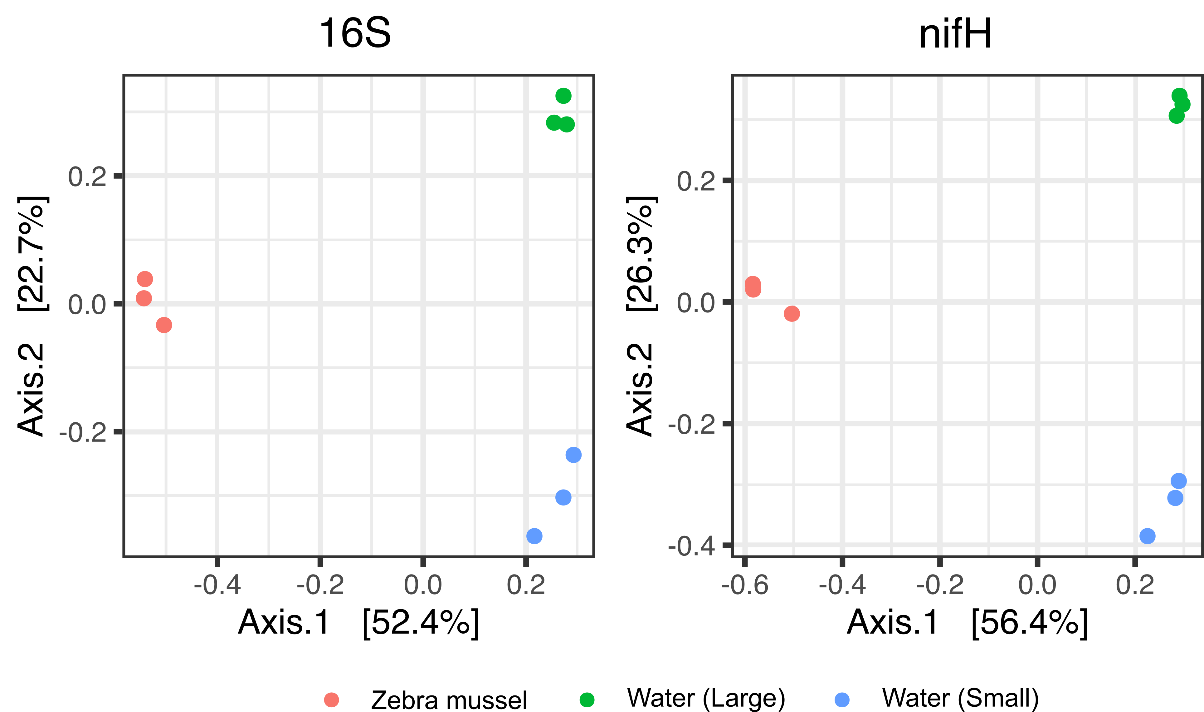


**A**

**B**

**Figure S4**. PCoA (Principal Coordinates Analysis) computed with Bray-Curtis distance calculated on ASV tables obtained for the 16S rRNA gene (panel A) and *nifH* gene (panel B) datasets. Each dot represents a sample.

**
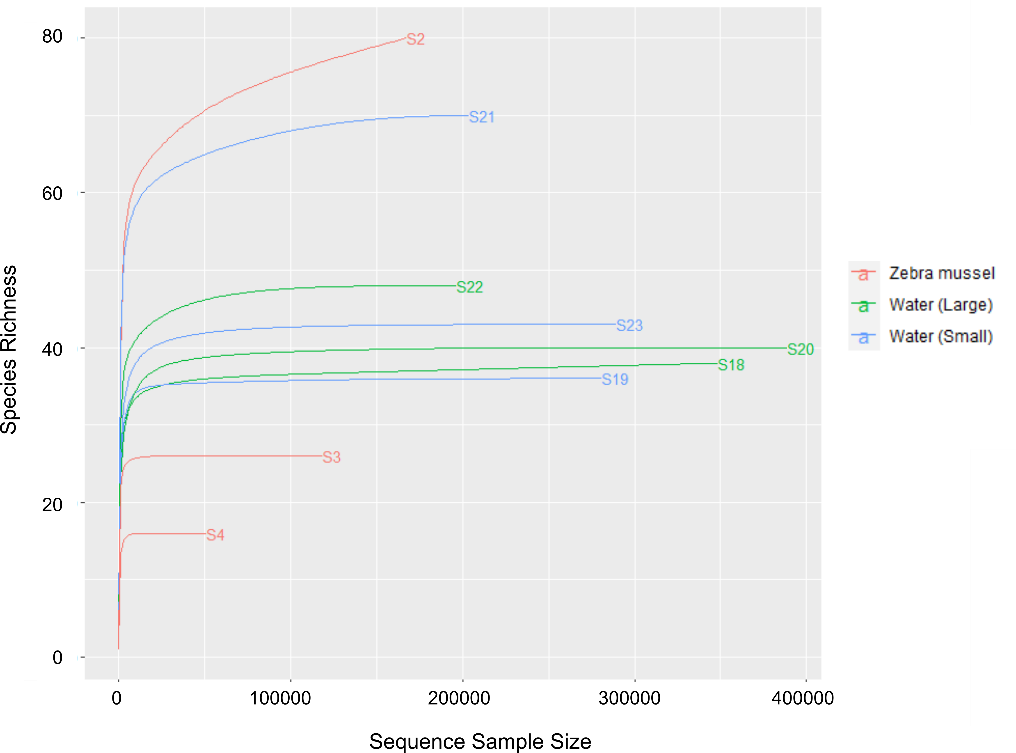
**

**Figure S5.**Rarefaction curves of the *nifH* samples analysed in this study.S2, S3 and S4 refer to zebra mussel samples. Samples S18, S20 and S22 refer to “Large” water samples, *i.e.*, water column particulate matter of size > 10 µm; samples S19, S21 and S23 refer to “Small” water samples, *i.e.*, water column particulate matter of size 0.22-10 µm.

*
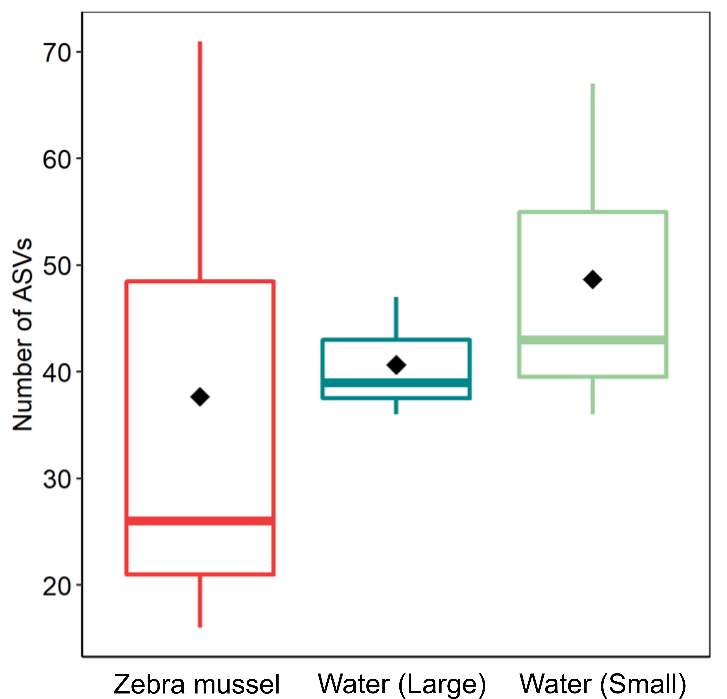
*

**Figure S6.**Boxplots showing*nifH* ASV richness of samples of zebra mussels and water column particulate matter of size > 10 µm (Large) and 0.22 - 10 µm (Small).
